# Periostin is a negative prognostic factor and promotes cancer cell proliferation in non-small cell lung cancer

**DOI:** 10.18632/oncotarget.25435

**Published:** 2018-07-27

**Authors:** Toshimasa Okazaki, Keiichi Tamai, Rie Shibuya, Mao Nakamura, Mai Mochizuki, Kazunori Yamaguchi, Jiro Abe, Satomi Takahashi, Ikuro Sato, Akira Kudo, Yoshinori Okada, Kennichi Satoh

**Affiliations:** ^1^ Division of Cancer Stem Cell, Miyagi Cancer Center Research Institute, Natori, Japan; ^2^ Division of Molecular and Cellular Oncology, Miyagi Cancer Center Research Institute, Natori, Japan; ^3^ Department of Thoracic Surgery, Miyagi Cancer Center, Natori, Japan; ^4^ Department of Pathology, Miyagi Cancer Center, Natori, Japan; ^5^ Department of Biological Information, Tokyo Institute of Technology, Yokohama, Japan; ^6^ Department of Thoracic Surgery, Institute of Development, Aging and Cancer, Tohoku University, Sendai, Japan

**Keywords:** periostin, lung cancer, cell proliferation, prognostic factor, prognosis

## Abstract

Periostin is a matricellular protein that is secreted by fibroblasts and interacts with various cell-surface integrin molecules. Although periostin is known to support tumor development in human malignancies, little is known about its effect on lung-cancer progression. We here demonstrate that periostin is a negative prognostic factor that increases tumor proliferation through ERK signaling in non-small cell lung carcinoma. We classified 189 clinical specimens from patients with non-small cell lung-cancer according to high or low periostin expression, and found a better prognosis for patients with low rather than high periostin, even in cases of advanced-stage cancer. In a syngenic implantation model, murine Ex3LL lung-cancer cells formed smaller tumor nodules in periostin^−/−^ mice than in periostin^+/+^ mice, both at the primary site and at metastatic lung sites. An *in vitro* proliferation assay showed that stimulation with recombinant periostin increased Ex3LL-cell proliferation. We also found that periostin promotes ERK phosphorylation, but not Akt or FAK activation. These findings suggest that periostin represents a potential target in lung-cancer tumor progression.

## INTRODUCTION

Lung cancer is the most common cause of cancer deaths world-wide [1]. Non-small cell lung cancer (NSCLC) accounts for more than 80% of all lung cancer. Less than 10% of patients with stage IV NSCLC survive five years after the diagnosis, while the 5-year survival rate of those with stage IA is as high as 80%. Multimodal treatment, including surgery, chemotherapy, and radiotherapy, does not satisfactorily improve the prognosis [2, 3]. NSCLC treatment strategies are changing with the development of therapies that target specific molecules, especially epidermal growth factor (EGF) receptor tyrosine kinase inhibitors, anaplastic lymphoma kinase inhibitors, and immune checkpoint inhibitors [4]. The prognosis for NSCLC depends on factors such as the patient's overall health and the stage and pathological type of NSCLC. Although the prognosis for NSCLC has improved, new prognostic markers and therapeutic strategies are still needed [5].

Periostin, a secreted matrix N-glycoprotein that lacks a transmembrane domain, has an NH_2_-terminal signal peptide sequence, internal homologous repeats, a cysteine-rich domain, and a hydrophilic COOH-terminal domain [6]. Periostin binds integrins, initiating cross-talk between the integrins and receptor tyrosine kinases such as EGF receptor at the plasma membrane, thereby co-activating the Akt and focal adhesion kinase (FAK) cell-signaling pathway that modulates cell motility, proliferation, and survival [7–12]. Periostin also enhances the growth of gastric cancer-cell lines accompanied by the activation of extracellular signal-regulated kinase (ERK) [13].

Several studies indicate that periostin overexpression or elevated serum periostin is associated with poor patient outcomes [14–19]. Recent studies suggest an association between high serum periostin and a poor prognosis in NSCLC [20, 21]. However, periostin's involvement in the progression of NSCLC is not fully understood.

In this study, we investigated the role of periostin in NSCLC using periostin-knock out mice and demonstrated that fibroblast-secreted periostin is crucial for NSCLC development, and especially for NSCLC-cell proliferation.

## RESULTS

### Periostin expression is correlated with a poor prognosis in lung cancer

**Figure 1:**
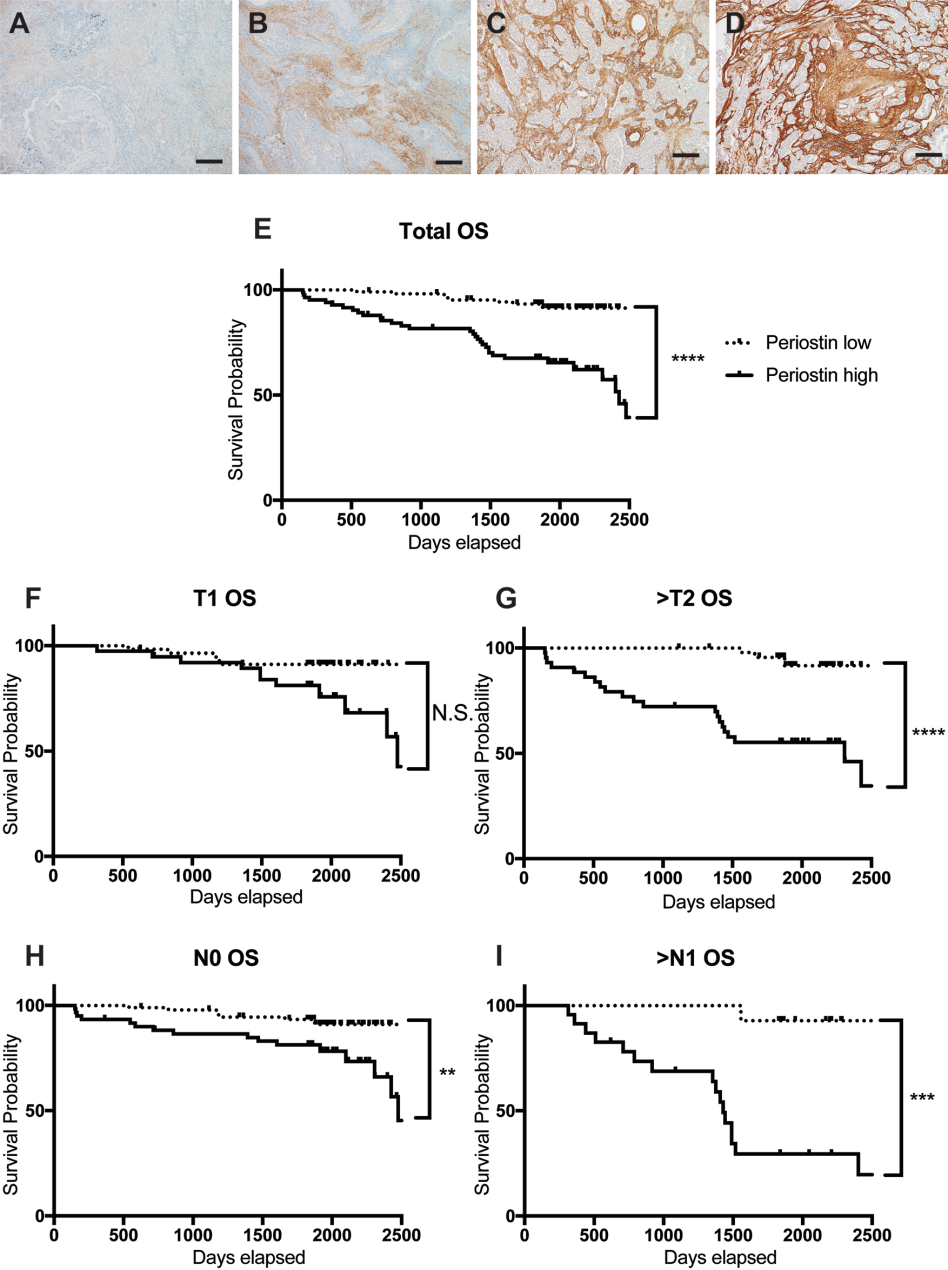
Periostin expression in human lung cancer **(A-D)** Representative results of IHC for periostin in clinical specimens obtained from 189 lung cancer patients during surgical resection. Specimens were scored for the amount of anti-periostin staining, with scores of 0 (A) or 1 (B) considered low and scores of 2 (C) or 3 (D) considered high. Scale bar: 100 μm. (E-I) Survival curves for NSCLC according to IHC periostin-staining grades, for **(E)** total population and by population in **(F)** T1 stage (*n*=97), **(G)** T2, 3, or 4 (*n*=92), (H) N0 (*n*=152), and (I) N1 and N2 (*n*=37). The 5-year survival rate by low or high periostin level in each population was (E) 93.3% for low (*n*=106) and 67.5% for high (*n*=83); (F) 91.3% for low (*n*=58) and 81.3% for high (*n*=39); (G) 91.7% for low (*n*=48) and 55.3% for high (*n*=44); **(H)** 93.3% for low (*n*=92) and 81.3% for high (*n*=60); and **(I)** 92.8% for low (*n*=14) and 29.5% for high (*n*=23). N.S., not significant.

To investigate associations between periostin expression and clinical outcomes, we examined specimens from 189 cases of lung cancer by immunohistochemistry (IHC) using anti-periostin staining. Periostin expression in the stroma ranged from weak to strong, but was consistently absent in the tumor area (Figure 1A to 1D) in all specimens. Cases were classified as low periostin (106 cases) or high periostin (83 cases) based on IHC. Table 1 summarizes the clinical and pathological characteristics of the patients in the cases. Periostin expression was correlated with gender, smoking, tumor size, pathological N factor (pN), pleural invasion (pl), and blood-vessel invasion (v). We then introduced gender, smoking, pathological T factor (pT), pN, pl, lymphatic invasion (ly), v, and periostin into a Cox regression model for multivariate analysis of overall survival (Table 2), and found that pT, pN, and periostin were independent predictors of survival. Kaplan–Meier analysis of overall survival according to low or high periostin (Figure 1E to 1I) showed that the median survival was significantly shorter in the periostin-high group (Figure 1E). Interestingly, the survival times were also shorter for the high-periostin group when comparing patients with advanced-stage NSCLC (pT2-4 or pN1-2) (Figure 1G and 1I). These data indicated that periostin is a negative prognostic factor in NSCLC.

**Table 1:** Characteristics of patients with non-small cell lung cancer

Characteristics		Low periostin low n=106	High periostin n=83	*p* value
**Age**	**Mean±SD (range)**	**67.47±0.86 (42-82)**	**64.95±0.95 (40-80)**	**0.19**
	**<70 / ≧70**	**55 / 51**	**51 / 32**	
**Gender**	**M / F**	**51 / 55**	**55 / 28**	**0.01**
**Smoking**	**−/ +**	**58 / 48**	**30 / 53**	**0.01**
	**Adenocarcinoma**	**86**	**62**	
**Histology**	**Squamous cell ca**	**10**	**14**	**0.48**
	**Others**	**10**	**7**	
**Tumor size**	**Mean±SD (range)**	**26.14±1.30 (7-75)**	**33.4±1.89 (9-98)**	**0.001**
	**T1**	**58**	**39**	
**T factor**	**T2**	**44**	**38**	**0.1**
	**T3-4**	**4**	**6**	
**N factor**	**N0**	**92**	**60**	**0.01**
	**N1-2**	**14**	**23**	
**pl**	**0**	**89**	**56**	**0.04**
	**1-3**	**17**	**27**	
**ly**	**0**	**103**	**79**	**0.47**
	**1**	**3**	**4**	
**v**	**0**	**96**	**59**	**0.0005**
	**1**	**10**	**24**	

Abbreviations: pl, pleural invasion factor; ly, lymphatic permeation; v, vascular invasion.

**Table 2:** Associations between survival rate and clinicopathological variables

			Univariate analysis^a^		Multivariate analysis^a^		
Variables		*n*	5-yr survival (%)	*p*	HR	95% CI	*p*
**Gender**	**M / F**	**106 / 83**	**74.7 / 92.6**	**0.006**	**1.36**	**0.47 - 4.07**	**0.57**
**Smoking**	**yes / no**	**88 / 101**	**73.3 / 93.0**	**0.003**	**1.37**	**0.51 - 4.10**	**0.55**
**Tumor size**	**30< / ≤30**	**75 / 114**	**71.1 / 90.1**	**0.001**	**1.45**	0.69 ^*^ 3.04	**0.33**
**pT**	**2-4 / 1**	**92 / 97**	**57.1 / 85.8**	**0.0009**	**3.15**	**1.22 - 7.82**	**0.02**
**pN**	**1-2 / 0**	**37 / 152**	**54.9 / 89.3**	**<0.0001**	**2.62**	**1.23 - 5.40**	**0.01**
**pl**	**1-3 / 0**	**44 / 145**	**69.4 / 86.5**	**0.03**	**1.72**	**0.76 - 4.22**	**0.2**
**ly**	**1 / 0**	**7 / 182**	**57.1 / 83.6**	**0.007**	**2.51**	**0.60 - 9.05**	**0.2**
**v**	**1 / 0**	**34 / 153**	**61.6 / 87.3**	**0.0003**	**1.35**	**0.63 - 2.76**	**0.43**
**Periostin**	**high / low**	**83 / 106**	**67.5 / 93.3**	**<0.0001**	**5.99**	**3.12 - 18.00**	**<0.0001**

^a^ Univariable analysis by log-rank test; multivariable analysis by Cox's proportional hazards model.

Abbreviations: pT, pathological T factor; pN, pathological N factor; pl, pleural invasion factor; ly, lymphatic permeation; v, vascular invasion.

### Absence of periostin expression reduces primary and metastatic tumor growth

**Figure 2:**
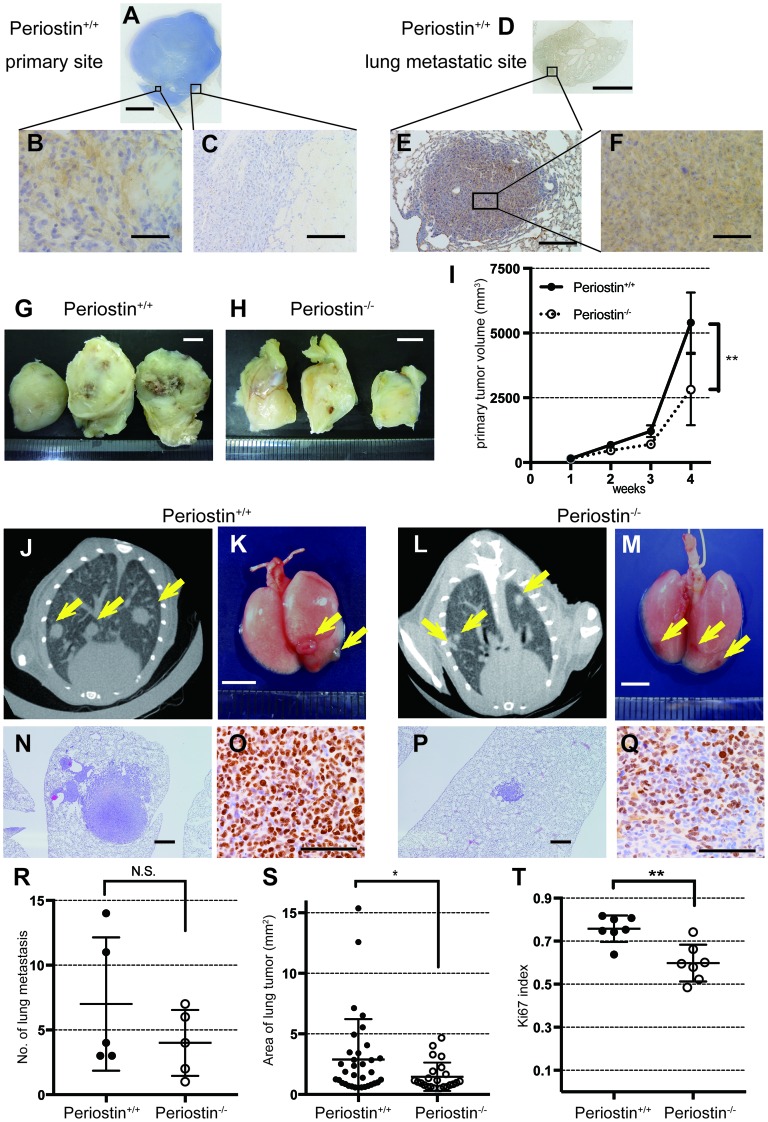
Periostin promotes lung-cancer proliferation *in vivo* Ex3LL cells were inoculated into the left thigh and observed for 4 weeks, followed by IHC for periostin of primary tumors and lung metastases. **(A)** Loupe images show a primary tumor generated in the left thigh and **(D)** metastasis in the lung in a periostin ^+/+^ mouse. Magnified views are shown in **(B)**, **(C)**, **(E)**, and **(F)**. **(G)** Tumors resected 4 weeks after inoculation from a periostin^+/+^ mouse and **(H)** a periostin^−/−^ mouse, and **(I)** average tumor volumes. **(J** and **L)** CT images of the lung from periostin^+/+^ and **(L)** periostin^−/−^ mice 4 weeks after cell implantation. Arrows indicate metastatic tumor nodules. **(K)** Macroscopic images of lungs resected 4 weeks after inoculation from periostin^+/+^ and **(M)** periostin^−/−^ mice; arrows indicate metastatic tumor nodules. **(N)** Microscopic images of the lung from periostin^+/+^ and **(P)** periostin^−/−^ mice, showing metastatic lesions stained with H&E. **(O)** IHC for Ki67 in metastatic lesions in the lungs, showing the **(R)** average number of metastatic nodules and **(S)** area of metastasis, determined from CT images. **(T)** The Ki-67 index (counted in at least three fields) in metastatic lesions in the periostin^+/+^ and periostin^−/−^ lung. Scale bar: 5 mm (A, D, G, H, K, and M), 50 μm (B and F), and 100 μm (C, E, and N to **Q**). N.S., not significant. The data are represented as mean ± SD.

Because periostin expression was correlated with tumor size and invasiveness, we investigated whether periostin affects NSCLC proliferation and metastasis. We implanted murine Ex Lewis Lung Carcinoma (Ex3LL) cells into the left thigh muscle of periostin^−/−^ and periostin^+/+^ mice and allowed four weeks for tumors to develop in the tissue, after which the metastatic nodules in the lungs were examined histologically, macroscopically, and radiologically. In the primary site, periostin was detected in fibroblasts adjacent to cancer cells in periostin^+/+^ mice, while no periostin-positive fibroblasts were observed in periostin^−/−^ mice (Figure 2A, 2B, Supplementary Figure 1A, and 1B). There was almost no fibrotic capsule at the primary lesion in either mouse strain (Figure 2C and Supplementary Figure 1C). Metastatic nodules formed in the lungs of both periostin^−/−^ and periostin^+/+^ mice, and the staining pattern of periostin was similar in the primary tumors and metastatic nodules in both mouse strains (Figure 2D to 2F and Supplementary Figure 1D to 1F). Examination of the size and number of primary and metastatic tumor lesions revealed that the primary tumors were smaller in periostin^−/−^ mice (2,947 ± 544.5 mm^3^) than in periostin^+/+^ mice (5,656 ± 461 mm^3^; Figure 2G to 2I). The number of nodules, counted by macroscopy and on CT images, did not differ significantly between periostin^−/−^ (4.00 ± 1.14) and periostin^+/+^ mice (5.29 ± 1.94) (Figure 2J to 2M and 2R). Measurement of the area of each lung metastasis site at the maximum plane in CT images revealed that the lesions were significantly smaller in periostin^−/−^ mice (1.32 ± 0.24 mm^2^) than in periostin^+/+^ mice (2.89 ± 0.47 mm^2^) (Figure 2J, 2L, 2N, 2P, and 2S). In addition, staining of the lung tissues with anti-Ki67 revealed that the Ki67 index (the ratio of Ki67-positive cells to nuclei) was significantly lower in periostin^−/−^ mice (0.598 ± 0.047) than in periostin^+/+^ mice (0.758 ± 0.023) (Figure 2O, 2Q, and 2T).

### Ex3LL supernatant promotes periostin expression in NIH/3T3 cells

**Figure 3:**
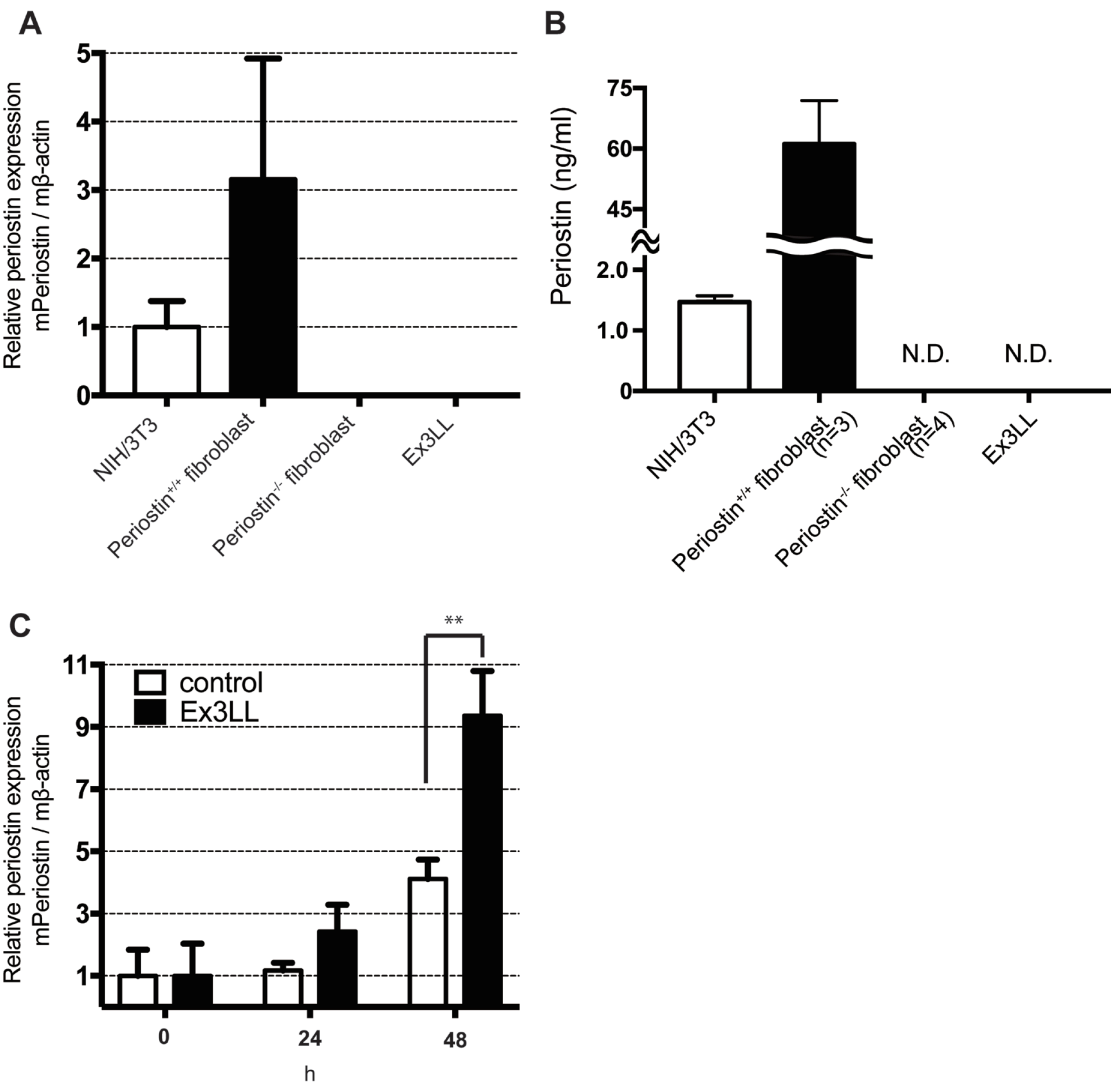
Ex3LL supernatant promotes periostin expression in NIH/3T3 cells **(A** and **B)** Periostin expression in NIH/3T3 cells, periostin^+/+^ fibroblasts, periostin^−/−^ fibroblasts, and Ex3LL cells at steady state, determined by real-time PCR (A, *n*=3) and ELISA (B) N.D, not detected. **(C)** NIH/3T3 cells were cultured with the conditioned medium of NIH/3T3 (control) or Ex3LL (Ex3LL) cells, and the periostin expression was measured by real-time PCR (*n*=3).

To investigate the relationship between cancer cells and the secretion of periostin from adjacent fibroblasts, we measured the periostin expression from fibroblasts cultured in medium conditioned by cancer cells. Ex3LL cells secreted almost no periostin compared to cells from the mouse NIH/3T3 fibroblast line or fibroblasts established from mouse lungs (Figure 3A and 3B). NIH/3T3 cells were cultured for 48 h in supernatant from Ex3LL cells (RPMI containing 1% fetal bovine serum) or with NIH/3T3-cell supernatant as a negative control. Cells cultured in the Ex3LL supernatant secreted more periostin than control cells (Figure 3B). These data suggested that cancer cells promote the secretion of periostin from adjacent fibroblasts.

### Periostin promotes cell proliferation *in vitro*

**Figure 4:**
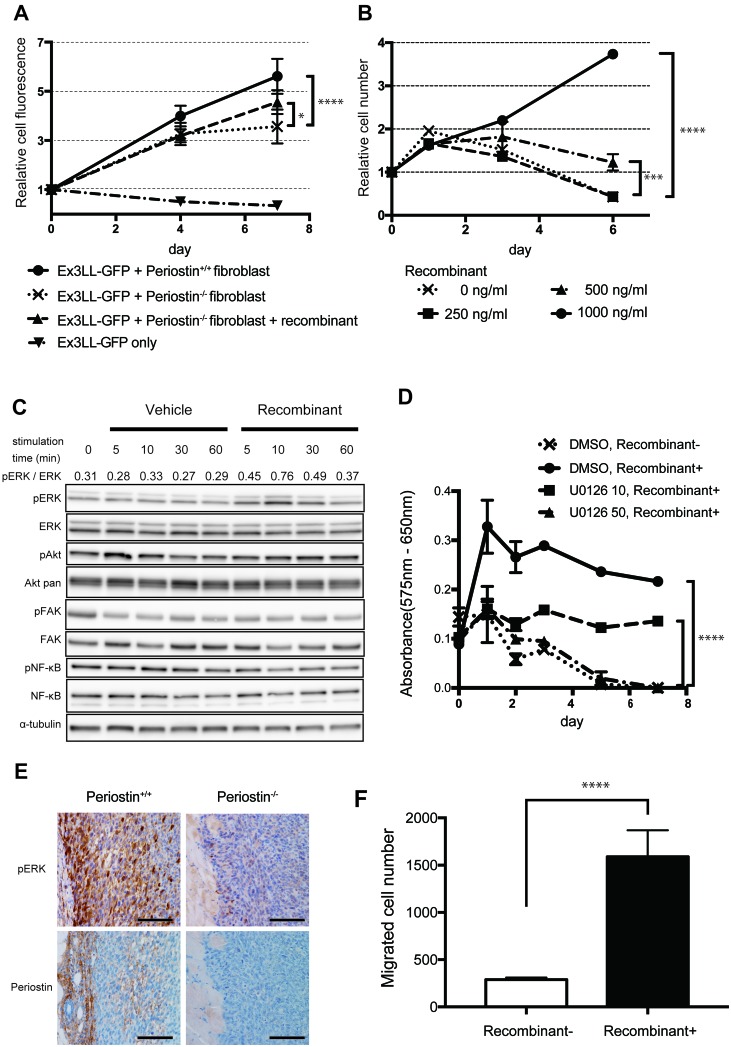
Periostin promotes Ex3LL-cell proliferation and intracellular signaling *in vitro* **(A)** GFP fluorescence in Ex3LL-GFP cells co-cultured with periostin^−/−^ or periostin^+/+^ fibroblasts (*n*=5). Values on the Y-axis are normalized to the value on day 0. **(B)** Effect of periostin stimulation on Ex3LL proliferation, assayed by MTT (*n*=5). Values on the Y-axis are normalized to the value on day 0. **(C)** Ex3LL cells were incubated for 24 h with medium containing 0.1% FBS and then treated with 1,000 ng/ml periostin for 5, 10, 30, or 60 min. The presence of ERK, AKT, FAK, and NF-κB and their phosphorylated forms [pERK (Thr202/Tyr204), pAKT (Thr308), pFAK (Tyr925), and p-NF-kB (Ser536)] in the Ex3LL cells was assessed by western blotting. Ratios of pERK to total ERK, based on band density analyzed with ImageJ software, are shown. **(D)** Ex3LL cell proliferation measured by MTT assay. Cells were incubated for 24 h with medium containing 0.1% FBS, and then treated with recombinant periostin with or without U0126. ^****^P < 0.001 **(E)** IHC for pERK and periostin in primary tumors in periostin^+/+^ and periostin^−/−^ mice. Scale bar: 100 μm. **(F)** Ex3LL cells in 0.1% FBS with or without recombinant periostin were subjected to a two-chamber assay for cell migration.

To confirm the effect of periostin on cancer-cell proliferation, we assayed Ex3LL-cell proliferation *in vitro*. Because periostin was secreted mainly from fibroblasts (Figure 3A), we established fibroblast cell lines from the lungs of periostin^−/−^ and periostin^+/+^ mice and confirmed the presence of the fibroblast marker CD140a by FACS (Supplementary Figure 2A). We also confirmed the secretion of periostin from fibroblasts (Supplementary Figure 2B) We then investigated the effect of interactions between these lung fibroblasts and cancer cells in a co-culture assay. On day 7, cultures containing periostin^−/−^ fibroblasts had fewer Ex3LL cells than cultures containing periostin^+/+^ fibroblasts (Figure 4A). This loss of cell proliferation was partially rescued by adding recombinant periostin (rPeriostin); rPeriostin was added every 48 h at the same concentration, and by day 6 the proliferation rate was higher in the rPeriostin-stimulated than in unstimulated cultures (Figure 4B). These data indicated that periostin secreted from fibroblasts directly promotes cancer-cell proliferation.

We next investigated how periostin promotes the proliferative ability of cancer cells. Since previous reports suggested that periostin promotes cell proliferation by activating ERK-, Akt/PKB-, and FAK-mediated signaling pathways, we analyzed the intracellular signaling in Ex3LL cells exposed to periostin. Periostin stimulation increased the phosphorylated ERK (pERK) level (Figure 4C), but did not affect the pAkt, pFAK, or pNF-κB levels. To determine whether ERK signaling affected the periostin-induced cell proliferation, we performed MTT assays on Ex3LL cells incubated with periostin and the MEK inhibitor U0126 (Supplementary Figure 4). The periostin-induced cell proliferation was clearly suppressed in the presence of U0126 (Figure 4D). IHC for pERK and periostin in specimens from periostin^−/−^ and periostin^+/+^ mice revealed that pERK was expressed in the periphery of the primary tumor, adjacent to the periostin-positive stroma, in the periostin^+/+^ mice. In contrast, pERK was expressed only weakly in periostin^−/−^ mice (Figure 4E). These data suggested that ERK signaling is a major downstream component of the periostin-related pathway in Ex3LL cells.

Since we obtained evidence that periostin was involved in lymph node metastasis (Tables 1 and 2) and the metastatic sites tended to decrease in periostin^–/–^ mice, we examined the Ex3LL cell migration ability by a two-chamber assay. We found more migrated cells in the periostin-treated samples than in the controls (Figure 4F). These data suggested that periostin plays critical roles not only in tumor cell proliferation, but also in the migration ability of tumor cells.

## DISCUSSION

In this study, we demonstrated that tumor growth was reduced at both primary and metastatic sites in periostin^−/−^ mice compared to periostin^+/+^ mice, although there was no difference in the number of metastatic nodules. Another study reported that subcutaneously injected 3LL cells produced larger tumors in periostin^−/−^ mice than in periostin^+/+^ mice due to impaired tumor capsule formation [22]. Since we observed only slight encapsulation of the primary tumors formed in the thigh of both periostin^−/−^ and periostin^+/+^ mice, we speculate that periostin predominantly affected tumor proliferation in our study. When we injected Ex3LL cells into the tail vein of periostin^−/−^ and periostin^+/+^ mice, there was no difference in the number of metastatic lung nodules between the two groups (Supplementary Figure 3). These data suggest that periostin is involved in cancer-cell proliferation but not in colonization ability. In contrast, another report found that periostin is a key factor for metastatic colonization in breast cancer through the maintenance of cancer stem cells [23]. Such cancer stem cells or similar cells might not be present in the Ex3LL cell line, which is a subclone derived from 3LL cells [24] and might be more homogeneous. Further study is needed to determine whether periostin gives lung cancers the ability to maintain cancer stem cells and to colonize.

In this study, we demonstrated that periostin stimulation increased the pERK level in Ex3LL cells. Other reports suggest that periostin supports growth in gastric cancer cells through ERK activation [13], and that ERK signaling occurs downstream of periostin in lung cancer [25] and pancreatic cancer [26]. These data are consistent with our present study. In contrast, the involvement of the Akt/PKB and FAK pathways downstream of periostin has been reported previously [7, 8, 27, 28] but was not identified in the present study. This difference might be due to cellular context, such as differences in intracellular signaling in human or murine lung-cancer lines.

High serum periostin has been identified as a factor for poor prognosis in lung cancer [14, 15, 20, 21], and periostin overexpression in NSCLC tissue, identified by IHC, is also correlated with a poor prognosis [16–19]. In the present study, survival times were better for the low-periostin group than the high-periostin group, even for patients with lymph-node metastasis. These data strongly suggest that periostin is an independent predictor for prognosis in NSCLC. A recent study suggested that periostin deficiency decreases the immunosuppressive functions of myeloid-derived suppressor cells (MDSCs) during tumor progression, impairing the MDSC-promoted lung metastasis of breast-tumor cells [29]. Our study demonstrated that interaction between fibroblasts and cancer cells is important for the secretion of periostin from fibroblasts, which is compatible with reports for pancreatic cancer and renal cell carcinoma [30–32]. Cytokines from cancer cells, especially transforming growth factor-β (TGF-β), increase the periostin secretion from fibroblasts [6, 33]. TGF-β, which is known to activate tumor metastasis and invasion [34], might contribute to the poor prognosis in periostin-high patients. Thus, periostin-related pathways may play multiple roles in cancer malignancy and poor clinical outcomes.

In summary, our results demonstrated that high periostin expression is a strong prognostic factor in lung cancer, and that periostin secreted by adjacent fibroblasts may promote lung cancer proliferation and invasion. Periostin-related pathways are an attractive therapeutic target for extending survival in NSCLC.

## MATERIALS AND METHODS

### Ethics statement

This study was conducted according to the principles expressed in the Declaration of Helsinki, and was approved by the Ethics Committees at the Miyagi Cancer Center Research Institute (Natori, Japan). All animal experimental protocols (MCC-AE-2016-7) were approved by the Miyagi Cancer Center Animal Care and Use Committee.

### Patients and tissue specimens

This study included 189 consecutive NSCLC patients who underwent complete surgical resection at Miyagi Cancer Center between January 2009 and December 2011. Complete resection was defined as either lobectomy or more extensive resection with systematic mediastinal lymph-node dissection, and an absence of residual cancer both macroscopically and histologically. Patients who underwent exploratory thoracotomy or only partial resection of the main lesions were excluded. Patients with metastases from non-lung primary cancers or with double operations were also excluded. All cases were classified histopathologically according to the World Health Organization Classification of Tumors 2004 criteria, and the stage was classified according to the 7th edition of the UICC TNM staging system. Adjuvant chemotherapy was chosen according to the patient's general condition and preference: some patients with stage II and III disease were treated with platinum-based doublet regimens, and patients with stage IA or IB with a tumor size >20 mm were treated with oral tegafur/uracil. All patients were followed on an outpatient basis for five years after surgery; each patient underwent a physical check-up, chest computed tomography, and laboratory testing at least every 6 months and a systemic examination every 12 months. We reviewed the patients’ medical records for the following clinicopathological factors: age, gender, pathological T and N (pT and pN) classification, histological type, pleural invasion, and lymphovascular invasion. Overall survival was defined as the interval between the date of surgery and the date of death or last contact, respectively. All patients gave written informed consent for inclusion in the study.

### Immunohistochemistry

IHC was performed on 3-μm sections of human lung-cancer tissue using a Ventana Discovery automation system (Roche, Basel, Switzerland) with rabbit polyclonal anti-periostin antibody (ab14041, 1:2000, Abcam, Cambridge, UK) or anti-Ki67 (Clone 30-9, Roche) according to the manufacturers’ instructions. Phospho-ERK (pERK) was detected with anti-phospho-p44/42 MAPK (ERK1/2, Thr202/Tyr204) (20G11, 1:400, Cell Signaling Technology, Danvers, MA, USA) according to the manufacturer's protocol.

### Ki67 index calculation

The Ki67 index was calculated as the ratio of Ki67-positive cells to nuclei. Nuclei and Ki67-positive cells were counted with ImageJ software (National Institutes of Health, Bethesda, MD, USA).

### IHC periostin grading

The level of periostin IHC activity was scored semiquantitatively (0 to 3) according to the periostin-positive density and area in the stroma of the carcinoma. Specimens were scored independently by two cancer pathology experts in a blinded manner. We defined a score of 0 or 1 as low periostin, and a score of 2 or 3 as high periostin.

### Animals

C57BL/6 periostin-knockout (periostin^−/−^) mice were generated by Cre recombination as described previously [35]. C57BL/6 periostin wild type (periostin^+/+^) mice were used as a control.

### Cell lines

The mouse Ex Lewis Lung Carcinoma (Ex3LL) cell line, which is derived from the 3LL line and has enhanced lung-metastasis ability, was obtained from the Japanese Collection of Research Bioresources Cell Bank, National Institutes of Biomedical Innovation, Health and Nutrition (Osaka, Japan). The NIH/3T3 mouse fibroblast cell line was obtained from RIKEN BioResource Center (Tsukuba, Japan). Ex3LL cells were maintained in Roswell Park Memorial Institute Medium-1640 (RPMI-1640, Wako, Japan) supplemented with 10% fetal bovine serum (FBS, Corning, Corning, NY, USA) and penicillin–streptomycin (100 U/0.1 mg/mL) (Nacalai Tesque, Kyoto, Japan). NIH/3T3 cells were maintained in Dulbecco's Modified Eagle's Medium (DMEM, Wako) supplemented with 10% FBS and 1% penicillin–streptomycin. Cells were incubated in a humidified incubator at 37°C with 5% CO_2_. To reproduce a lung-cancer niche, we established heterogeneous primary fibroblasts from the lungs of periostin^−/−^ and periostin^+/+^ mice. The lungs were removed, washed in calcium-free PBS, cut into small fragments under aseptic conditions, and finely minced with a sterile scalpel. The minced lung tissue was incubated in the RPMI-1640 supplemented with 10% FBS and penicillin–streptomycin until the fibroblasts formed colonies (one week). The fibroblasts were passaged at least 10 times *in vitro* before being used in experiments.

### Implantation of cancer cells

Ex3LL cells (1×10^6^ in 100-mL PBS) were implanted into the left thigh muscle. Tumor formation was monitored weekly. Four weeks after implantation, the number of metastatic lung nodules was counted by micro X-ray computed tomography with a Cosmo Scan GX (Rigaku, Tokyo, Japan); the images were interpreted on OsiriX (Pixmeo SARL, Bernex, Switzerland) by a pulmonologist. The mice were then euthanized, and the primary tumors were weighed. The lungs were fixed with 10% formalin neutral buffer solution, and metastatic lung nodules were counted macroscopically. In the tail-vein assay for metastasis, Ex3LL cells (8×10^5^ in 200 mL PBS) were injected into the tail vein. Three weeks after the injection, the lungs were harvested and fixed in 10% formalin neutral buffer solution, and the lung nodules were counted macroscopically.

### Flow cytometry

To confirm that the primary cells isolated from mouse lungs contained fibroblasts, the cells were stained with specific antibodies for 30 min at 4°C, washed twice, and analyzed using a FACSCanto II (BD Biosciences, Franklin Lakes, NJ, USA). The antibodies used were: anti-mouse APC-CD140a antibody (clone APA5, BioLegend, San Diego, CA, USA) and anti-mouse FITC-CD326 antibody (clone G8.8, BioLegend). In addition, 7-AAD staining (Merck Millipore, Billerica, MA, USA) was used to exclude dead cells.

### Quantitative real-time PCR

Total RNA was extracted from NIH/3T3 or Ex3LL cells using the RNeasy Mini Kit (Qiagen, Valencia, CA, USA), and complementary DNAs (cDNAs) were synthesized from 1.0 μg of total RNA with the PrimeScript II cDNA Synthesis Kit (Takara Bio, Shiga, Japan) according to the manufacturers’ protocols. Real-time PCR was performed using Brilliant III Ultra-Fast SYBR Green QPCR Master Mix (Agilent Technologies, Santa Clara, CA, USA). β-actin was used as an endogenous reference gene. The following primer sequences were used: for mouse periostin, F 5′- cgaaggggacagtatctcca -3′ and R 5′- gcttcagagaggatgccaag - 3′; for mouse β-actin, F 5′- ctaaggccaaccgtgaaaag -3′ and R 5′ accagaggcatacagggaca -3′.

### Ex3LL-GFP cell line and co-culture assay

To establish the Ex3LL-GFP cell line, we first inserted the luciferase gene into pCSII-EF-IRES-GFP. This produced a pCSII-EF-Luc-IRES-GFP plasmid that was transfected into HEK293T cells together with three plasmids, pMDG, pMPL g/p PRE, and pRSV-rev, using FuGeneHD (Promega, Madison, WI, USA). Supernatants containing the recombinant lentivirus were used to infect Ex3LL cells. Stable cell lines were isolated by FACS Aria (Becton Dickinson) as GFP-positive cells. Periostin^−/−^ and periostin^+/+^ fibroblasts were cultured on 96-well tissue-culture plates until confluent, and were then treated with 10 μg/ml mitomycin C (Wako) for 2 h. The cells were washed twice, and 5×10^3^ Ex3LL-GFP cells were plated in 0.1 ml phenolsulfonphthalein-free RPMI-1640 medium supplemented with 1% FBS and 1% penicillin–streptomycin. Fluorescence was measured (excitation 485 nm, emission 515 nm) using a microplate reader (Synergy H1, BioTek, Winooski, VT, USA).

### Cell proliferation assay

Ex3LL cells (5×10^3^) were plated in 0.1 ml RPMI-1640 medium supplemented with 0.1% FBS and 1% penicillin–streptomycin in a 96-well plate (TPP, Switzerland). Various concentrations of recombinant mouse periostin (R&D, Minneapolis, MN, USA) were added. At the indicated times, MTT (3-(4,5-dimethylthiazole-2-yl)-2,5-diphenyl tetrazolium bromide) assay reagent (Roche) was added to each well, according to the manufacturer's protocol. Absorbance at 575 nm and 650 nm (background measurement) was determined using a plate reader (VersaMax ELISA Microplate Reader, Molecular Devices, Sunnyvale, CA, USA). At least three replicate wells were assayed for each condition, and the standard deviation was determined.

### Western blotting

Cells (1×10^5^) were washed once with PBS, lysed in 100 μl SDS loading buffer (100 mM Tris-Cl pH 6.8, 4% sodium dodecyl sulfate, 0.2% bromophenol blue, 20% glycerol, and 2% β-mercaptoethanol) and sonicated for 5 min. The samples were boiled for 5 min and subjected to SDS-PAGE, and the separated proteins were transferred onto a PVDF membrane (Merck Millipore) that had been incubated with 1:1,000-diluted primary antibody and then with HRP-conjugated anti-mouse or anti-rabbit antibody (Cell Signaling), as recommended by the manufacturers. Primary antibody binding was detected using a Clarity™ Western ECL substrate (Bio Rad, Hercules, CA, USA), and images were captured by a CCD camera (Fuji Film, Tokyo, Japan). The following primary antibodies were used: anti-pERK (20G11, Cell Signaling), anti-ERK (137E5, Cell Signaling), anti-phospho-Akt (Thr308) (C31E5E, Cell Signaling), anti-Akt pan (C67E7, Cell Signaling), anti-phospho-FAK (Tyr925) (Cell Signaling), anti-FAK (Cell Signaling), anti-phospho-NF-κB p65 (Ser536) (93H1, Cell Signaling), anti-NF-κB p65 (D14E12, Cell Signaling), and anti-α-tubulin (B-5-1-2, Santa Cruz Biotechnology, Dallas, TX, USA).

### Enzyme-linked immunosorbent assay (ELISA)

After a 48-h incubation, periostin levels in the culture supernatant of each fibroblast sample were determined by ELISA using the Mouse Periostin/OSF-2 Quantikine ELISA Kit (R&D, Minneapolis, MN, USA), according to the manufacturer's instructions. The periostin concentration was determined by differences in the absorbance at 450 nm and 570 nm (background measurement) using a plate reader (VersaMax ELISA Microplate Reader).

### Two-chamber assay

Cell migration was assessed using a two-chamber assay with a cell-culture insert (8-μm pore size, BD Biosciences, San Jose, CA, USA) in 24-well plates. Ex3LL cells were starved in medium containing 0.1% FBS overnight, treated with 10 μg/ml mitomycin, and plated in each insert (5×10^4^) in medium containing 0.1% FBS. The bottom well contained medium with 0.1% FBS and periostin (0 or 1000 ng/ml). After 24 h, the bottom of the insert was stained with Diff-Quick (Dade Behring Inc., Newark, DE, USA), and the cells that had migrated through the membrane to the lower surface were counted.

### Statistical analysis

Statistical analyses were performed using GraphPad Prism version 7.0a (GraphPad Software, La Jolla, CA, USA). Differences between two groups were analyzed by unpaired *t*-test or Pearson's chi-square test. The overall survival rate was determined by Kaplan–Meier analysis and evaluated by log-rank test. For multivariate analyses of significant factors identified by univariate analysis, we used Cox's proportional hazards model and JMP Pro 13.1.0 software (SAS, Cary, NC, USA). A *p* value < 0.05 was considered significant.

## SUPPLEMENTARY MATERIALS FIGURES



## Abbreviations

3LL: Lewis Lung Carcinoma
7-AAD: 7-Amino-Actinomycin D
APC: allophycocyanin
cDNA: complementary deoxyribonucleic acid
CT: computed tomography
EGF: epidermal growth factor
ELISA: enzyme linked immunosorbent assay
ERK: extracellular signal-regulated kinase
FACS: fluorescence activated cell sorting
FAK: focal adhesion kinase
FITC: fluorescein isothiocyanate
GAPDH: glyceraldehyde 3-phosphate dehydrogenase
GFP: green fluorescent protein
IHC: immunohistochemistry
MAPK: mitogen-activated protein kinase
MDSC: myeloid-derived suppressor cell
MTT: 3-(4,5-dimethylthiazole-2-yl)-2,5-diphenyl tetrazolium bromide
NSCLC: non-small cell lung cancer
OS: overall survival
PKB: protein kinase B
PVDF: polyvinylidene difluoride
TGF-β: transforming growth factor-β.
