# Supplementary material for: Periostin is a negative prognostic factor and promotes cancer cell proliferation in non-small cell lung cancer
Source: Oncotarget. 2018 Jul 27;9(58):31187–99. doi: 10.18632/oncotarget.25435 (PMC6101292; doi:10.18632/oncotarget.25435)
Supplement: Supplementary file 1 [file oncotarget-09-31187-s001.pdf]

Periostin is a negative prognostic factor and promotes cancer cell proliferation in non-small cell lung cancer

SUPPLEMENTARY MATERIALS

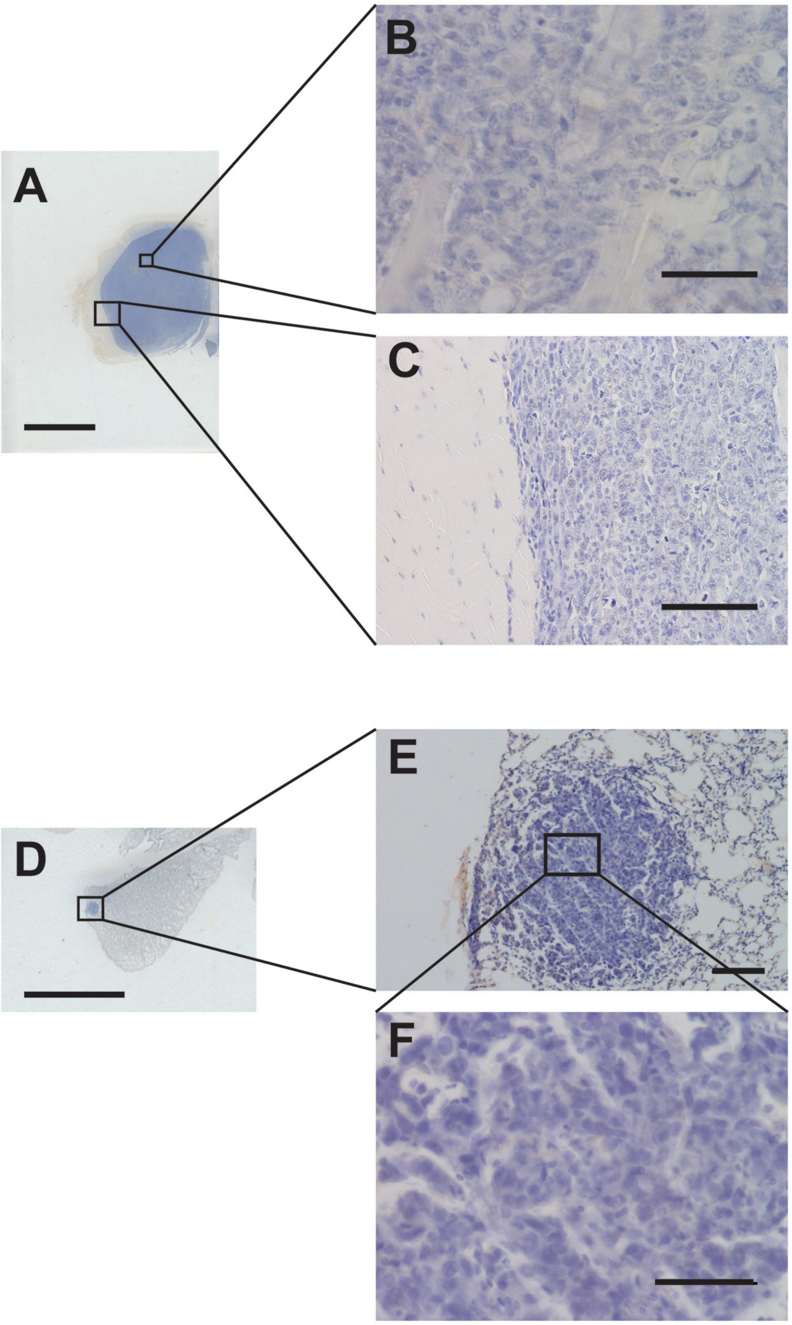

Supplementary Figure 1: Periostin in primary and metastatic tumor sites in periostin$^{-/-}$ mice. (A) Loupe images of a primary tumor in the left thigh and **(D)** and lung metastatic site in a periostin$^{-/-}$ mouse; magnified images are shown in **(B, C, E, and F)**. Scale bar: 5 mm (A and D), 50 µm (B and F), and 100 µm (C and E).

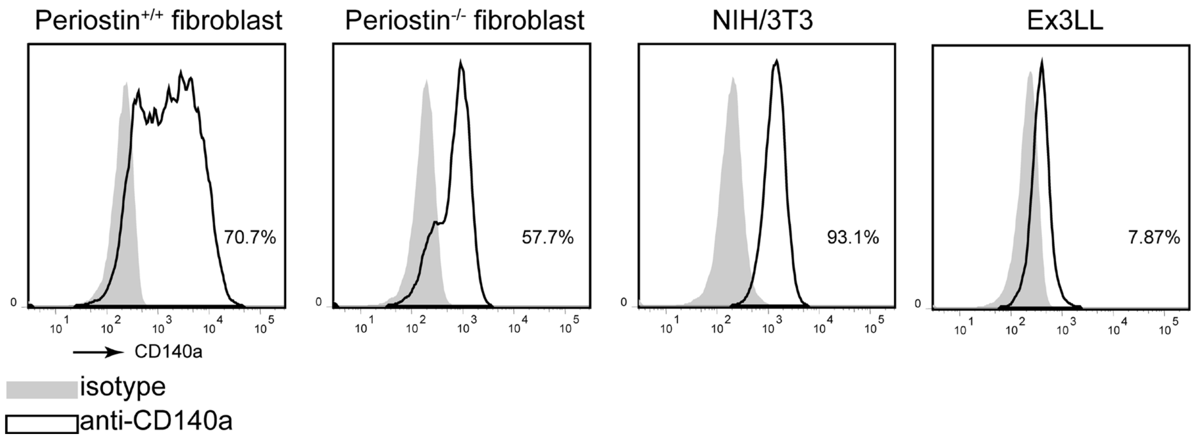

Supplementary Figure 2: Flow cytometry of periostin$^{+/+}$ and periostin$^{-/-}$ fibroblast lines. **(A)** Periostin$^{+/+}$ and periostin$^{-/-}$ fibroblast lines were stained with an anti-CD140a antibody and analyzed by flow cytometry. NIH/3T3 cells were used as a positive control, and Ex3LL cells as a negative control.

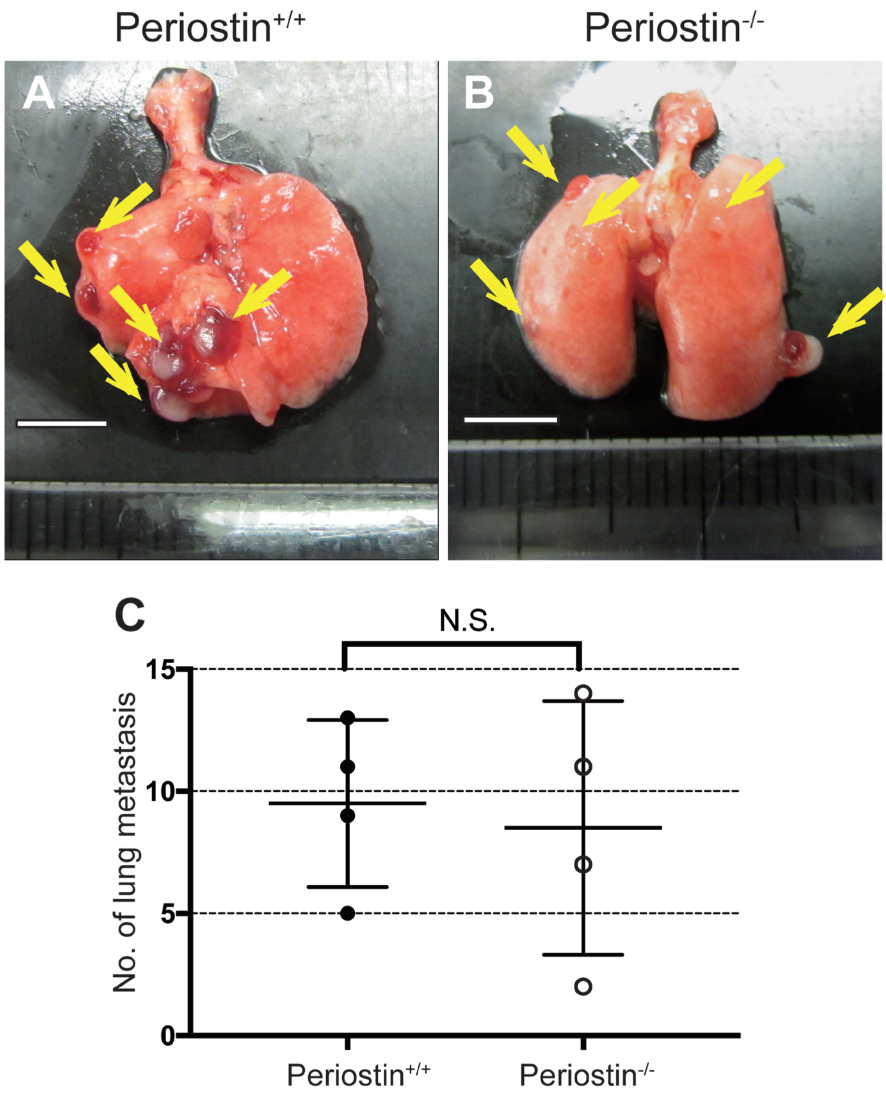

Supplementary Figure 3:Metastatic tumor nodules in the lung after injecting Ex3LL cells. (A) Macroscopic images show lungs of periostin$^{+/+}$ and **(B)** and periostin$^{-/-}$ mice examined 3 weeks after Ex3LL cells were injected into the tail vein. Arrows indicate metastatic tumor nodules. Scale bars: 5 mm. **(C)** Number of lung metastases in periostin$^{+/+}$ and periostin$^{-/-}$ mice. N.S., not significant.

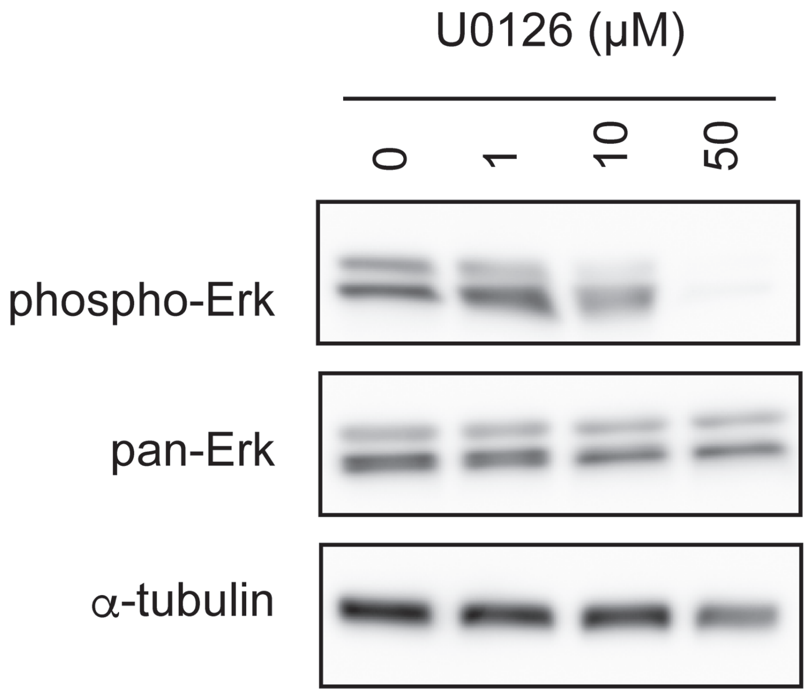

Supplementary Figure 4: Western blot analysis of ERK phosphorylation under the stimulation of U1026 (MEK inhibitor) in Ex3LL cells.
